# External frame distraction osteogenesis of the midface in the cleft patient

**DOI:** 10.4103/0970-0358.57193

**Published:** 2009-10

**Authors:** Syed Altaf Hussain

**Affiliations:** Cleft and Craniofacial Centre and Department of Plastic Surgery, Sri Ramachandra University, Chennai, India

**Keywords:** Cleft, istraction in clefts, Midface distraction

## Abstract

Distraction osteogenesis has established itself as an accepted form of treatment in the management of midface deficiency in cleft patients. However, it is well known that some amount of relapse is inevitable in patients who undergo this procedure. Like most surgical techniques, it has its specific indications, limitations, and complications. The problems are amplified in some patients because of severe fibrosis resulting from previous palate and lip operations. This article reviews treatment planning, pre- and postoperative orthodontic management, operative technique, and mechanics of distraction. It also discusses long-term changes following distraction and protocols to optimize the results and minimize complications.

## INTRODUCTION

Distraction osteogenesis of the long bones was first developed by Ilizarov in Russia in the late 1950s.[1] The first Russian language publication appeared in 1969.[2] A Medline search for distraction osteogenesis for midface advancement shows the earliest publication by Raschmiel *et al.* in the *British Journal of Plastic Surgery* in 1993.[3] After it was first described for children and young adults in 1997,[4] there have been several conflicting reports regarding its efficacy and advantages as well as short comings and high relapse rate[5–9] and hence the need for this technique to be used judiciously and with care.

It is well recognized that cleft lip and palate repair affects the three-dimensional growth of the maxilla. There are several reports of midface retrusion occurring in 25–70% cleft patients with up to 40% needing surgical treatment for the correction of midface deficiency.[10,11] The probable reasons for this are the fibrosis following surgical trauma and the intrinsic inability of the maxilla to grow normally.[12,13] This results in a midface deficiency with a Class III malocclusion and reverse overjet, which manifests during the initial growth phase of the child and becomes more pronounced as the child grows into adolescence. The anterior nasal spine and the pyriform margins which form the bony platform for the nose are posteriorly placed in relation to the skull base resulting in inadequate projection of the midface. There may be associated relative or absolute prognathism of the mandible. The alveolar arches may be collapsed and the teeth may be malpositioned or malrotated. In addition, some of these patients may have poorly repaired lip and palate with velopharyngeal incompetence as well as poor oral hygiene and dental caries. All these need to be addressed in stages, in addition to a successful distraction for a successful rehabilitation of these patients.

## INDICATIONS

The correction of the cleft midface deformity involves advancement of the midface by a Le Fort I level osteotomy. Our indications for distraction osteogenesis as against conventional orthognathic surgery are (a) patients who have not attained skeletal maturity, (b) patients requiring advancement of more than 7 mm of the maxilla alone, (c) patients with severe fibrosis of the lip and palate following multiple attempts at palate repair, and (d) those who have had a pharyngeal flap for VPI correction.

## PLANNING

Before the decision for distraction is taken, the patient has a joint consultation with the surgeon, orthodontist, and the speech pathologist. Dental arches are optimally prepared by alignment and decompensation of the teeth by the orthodontist. Fistulae, if they are present, are repaired. Alveolar bone grafting (ABG) should be done before distraction. In bilateral cases we complete ABG at least on one side and preferably on both sides before taking the patient up for distraction. If the alveolar arch is collapsed, it is expanded by an orthodontic device. We prefer to retain the orthodontic device during the period of expansion as it gives additional support to the hemi-maxillae and facilitates symmetrical distraction of the segments. We try and preserve the third molar, since if it does erupt, it may provide the much needed posterior occlusal edge to the advanced maxilla. It is essential to evaluate and record preoperative speech samples and if needed perform a nasoendoscopy. Since there is a possibility of imminent or mild VPI to become more prominent postoperatively, the patient needs to be informed about the implications of distraction on speech.,[14] The amount of advancement needed and the vector of distraction are established by mock surgery on the dental model. Alternatively, other advanced three-dimensional imaging and prediction software and stereolithography can also be used for planning.

## THE PRINCIPLES OF DISTRACTION OSTEOGENESIS

Distraction is the gradual stretching of callus after osteotomy of the bone. The callus responds to gradual stretching over few days by regenerating new bone. This has been shown by Ilizarov himself in his path-breaking study on reparative regeneration in dogs.[15] Several microscopic studies[16–18] have shown that after about 10 days of distraction (15th postoperative day), there is a central zone within the regenerate with proliferating mesenchymal cells and capillaries resulting from angiogenesis and a paracentral zone with large amount of wavy collagenous fibers. After 15 days of commencing distraction, there is appearance of mineralization and at 20 days, the trabeculae of the newly formed, delicate woven bone are oriented along the lines of distraction and become continuous with the nondistracted bone. The trabeculae get rimmed by osteoblasts and this is followed by remodelling. Another study with dispersive x-ray micro analysis[19] has shown progressive mineralization with an increase in the calcium and phosphorus content occurring from 3 weeks until 1 year. From these, one may conclude that the regenerated bone has physical and physiological properties unique to it and unless it is protected during this prolonged consolidation period, it is susceptible to relapse and deformation.

### Operative technique

The patient is anesthetized with oro-tracheal intubation. We prefer a south-going RAE tube which causes the least amount of interference to the surgeon. An upper sulcus incision with an adequate mucosal flange is made from 1st molar to opposite molar. Standard Le Fort I level I cuts are made on the maxillary bone taking care to stay at least 0.5 cm away from the roots of the teeth. This cut is started at a slightly higher (cranial) level at the pyriform margin medially and sloped down as it runs laterally and posteriorly to the zygomatic buttress to end just above the maxillary tuberosity. The nasal septum and the lateral nasal walls are osteotomized and disjunction at the pterygomaxillary junction is accomplished using appropriate osteotomes. Minimal amount of mobilization is made to ensure that the osteotomy is complete. We try to avoid an extensive down fracture since this may cause excessively floating segments.

**Figure 1:**
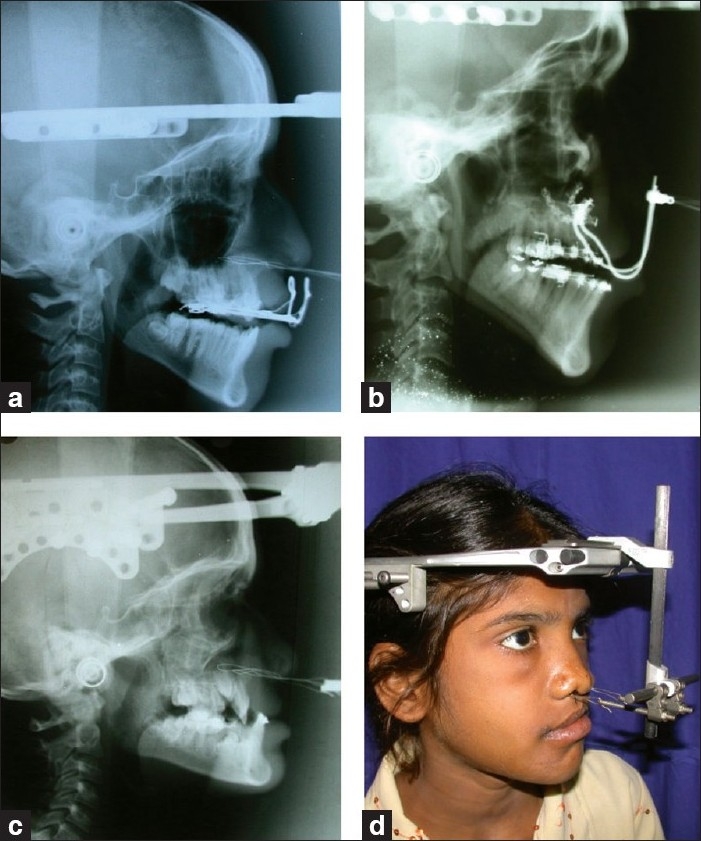
Top row: tooth-borne distraction device and the Leipzig plate. Bottom row: transpyriform wire and the external frame distractor

The distraction is accomplished by either an external or internal distractor. The commonest external device we use is the RED II distractor. This is a multiplanar extraoral device and unlike the intraoral device, the vector of distraction can be adjusted as the distraction proceeds. The major disadvantage is that it is cumbersome and needs to be worn for 8–10 weeks. The osteotomied segment is anchored to the device using 26 G stainless steel (SS) wires looped through a drill-hole made lateral to the paranasal buttress [Figure 1a]. These are brought out through skin at the nasal sill just medial to the alar base. We favour this method of anchorage because it is inexpensive and easily done. The other methods of anchoring are Leipzig plates[20] [Figure 1b] which attach to the maxilla. The advantages are that it has a better three-dimensional control on distraction compared to the transpyriform wires. The disadvantages are that it is expensive and needs a second surgery to remove it. The last means of anchorage is a tooth-based device which is an acrylic or metal splint moulded to the dental ridge. It has a curved metal rod exiting through the mouth to which the external distraction device is attached by a SS wire [Figure 1c]. It has the disadvantage of making oral hygiene difficult and may cause inadvertent extrusion of teeth or root exposure due to constant traction on the teeth. We only use this as a salvage device if other methods of anchorage fail.

Intraoral distraction devices are confined to the maxilla and are better tolerated by the patient. They are unidirectional and the vector cannot be changed once the device is inserted. They need precise preoperative vector planning with dental models and preferably stereolithographic models. The disadvantages are that they are expensive and also need a second surgery to remove them.

### Distraction

**Figure 2:**
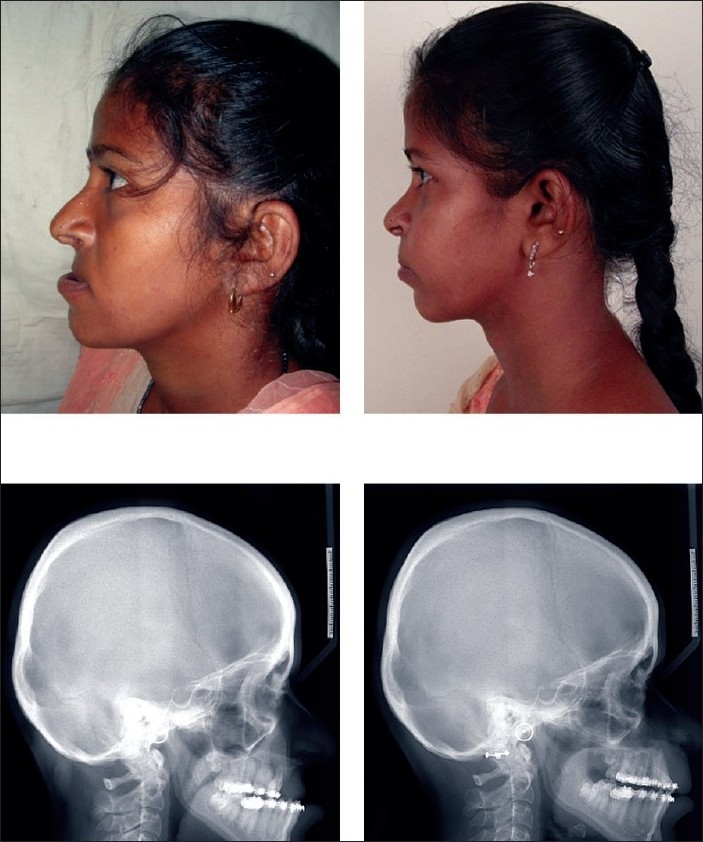
Top row: profile—pre- and postdistraction. Bottom row: lateral cephalogram—pre- and postdistraction

**Figure 3:**
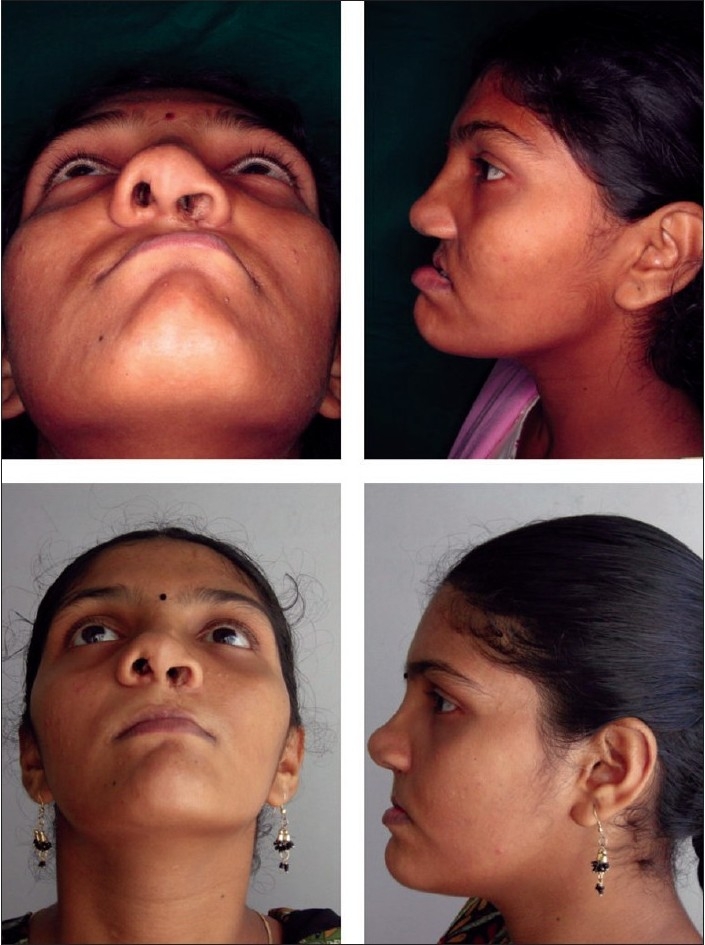
Top row: worm's eye view and profile—predistraction. Bottom row: worm's eye view and profile—postdistraction

**Figure 4:**
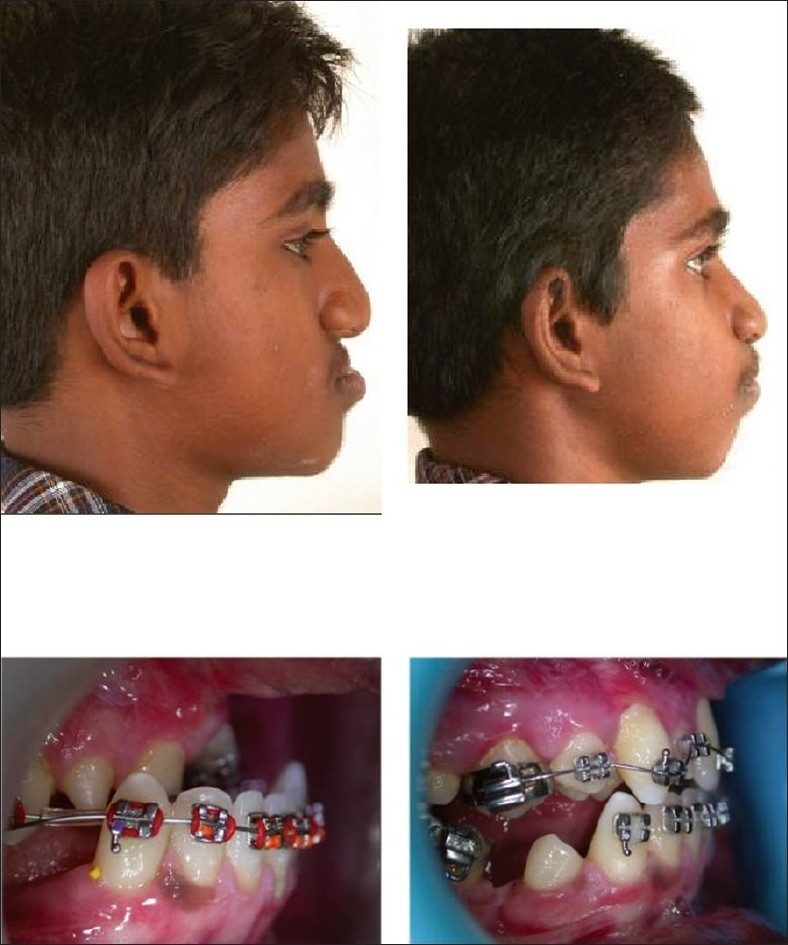
Top row: profile—pre- and postdistraction. Bottom row: occlusal view—pre- and postdistraction

**Figure 5:**
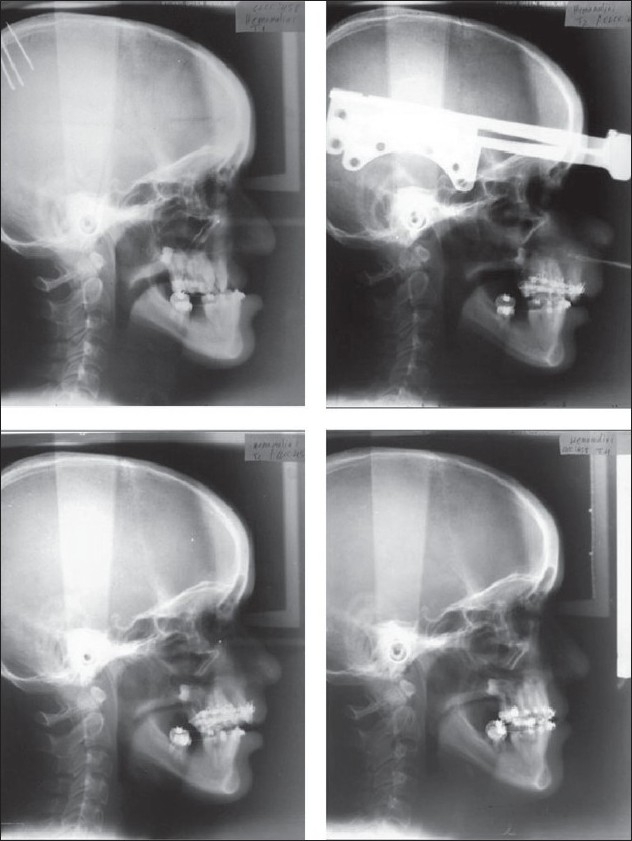
Top row: lateral cephalogram—before distraction and at the end of distraction.Bottom row: lateral cephalogram—at 6 months and 3 years postdistraction

Distraction vectors are planned preoperatively. The factors influencing the direction of the movement of the segments are the level of anchorage, the direction of the pull, and areas of resistance. The latter is determined by the soft tissue attachments of the maxilla and the scars of previous cleft repairs. The direction of the pull needs to be adjusted so that the anterior movement of the maxilla is along the occlusal plane. After a latency period of 5 days, distraction is commenced at the rate of 0.5 mm twice a day till the required advancement of the midface is achieved. Depending on the education and motivation level of the patient and the parent, this can be done either on an inpatient or outpatient basis. We prefer to keep the patient under close surveillance (at least twice a week) during the period of distraction. The distraction is closely monitored with the help of a lateral cephalogram repeated once a week during the activation phase. This is followed by a consolidation period of 2 months when the device is retained. After 8 weeks of consolidation are completed, the device is removed and postoperative orthodontics is commenced. Cephalograms are again repeated at 6, 12, and 36 months [Figures 2–4, 5].

The advancement is maintained by retention devices like the reverse-pull head gear and elastics to be worn at all times except when going to school or visiting people for 6 months following the distraction and at night for a further period of 12–18 months. In our series of 29 distractions using the external device, time away from school or work ranged from 1 to 6 months with a mean of 3 months.

## COMPLICATIONS AND THEIR MANAGEMENT

In addition to the complications associated with the osteotomy, like bleeding, there are a few which are unique to distraction. A common complication to be watched for is loosening of the pins holding the halo frame to the skull. This needs to be periodically tightened. We only hand-tighten these so that excessive pressure could be avoided. In rare instances, the pins could penetrate the inner table of the skull into the cranial cavity. This could occur because of mechanical factors or due to ischemic necrosis caused by long-standing pressure exerted by the pins. Incomplete osteotomy is the commonest cause of failure of distraction. The commonest sites of incomplete osteotomy are the posterior-medial part of the maxillary tuberosity, the vertical plate of the palatine bone in the lateral nasal wall, and the incomplete dysjunction of the pterygoids. These areas need to be addressed with care while completing the osteotomy. Incomplete osteotomy is characterized by a gradual increase in pain during distraction. If this is not identified early and reosteotomy is performed, it may result in failure of anchorage of the distraction device. If one side is not osteotomized, it may result in an asymmetrical advancement. The other causes of an asymmetrical distraction are improper adjustment of the device, asymmetric maxillary segments, and dense fibrosis involving one segment. This needs to be identified and the vector and the rate of distraction have to be adjusted diferentially. Occasionally, the trans-pyriform margin wires may cut through the bone. In some instances, this situation can be tackled by shifting traction to a tooth-borne device.

Consequences of the inevitable relapse are minimized by overcorrection. We routinely overdistract by 20–25% to compensate for this. Open bite is another complication which needs to be watched for as distraction progresses and may be minimized by varying the vector of distraction. If this is not effective, postoperative orthodontia with bite correction planes will be needed. In some cases, further osteotomies at a later date may be required.

In our series (29 patients), 1 patient had excessive peroperative bleeding. Three patients needed a repeat osteotomy and of these, two were for asymmetrical distraction due to an incomplete osteotomy on one side. Two had loosening of retention pins holding the halo frame of the external distraction device. Two patients had the traction wires inserted at the pyriform margin cutting through the bone during the course of distraction possibly due to resistance exerted by extensive fibrosis of the soft tissues from previous operations. Both these patients were shifted to a tooth-borne device. One of them had loosening of some teeth due to the device. All these complications could be identified and corrected during the course of distraction and consolidation. However, 1 patient had an asymmetrical final result and 2 out of the 29 patients had more than 50% relapse after the successful completion of distraction. One was due to failure of the mechanical device when the patient was at home during the distraction period and the second due to refusal of the patient to wear the device during the consolidation phase. Since the distraction in these cases was done before the completion of growth, in addition to relapse of the distracted maxilla, the remaining growth potential of the mandible may also contribute significantly to the long-term result. In an analysis of distractions in 20 adolescents in our series followed up for over 3 years, our orthodontic colleague[21] has reported an average of 20–25% relapse in the maxilla. In addition, it was found that the potential horizontal growth of the maxilla was reduced, while the vertical growth of maxilla and the antero-inferior growth of the mandible were unaffected [Figure 5]. Speech may be potentially affected in some cases because of the increase in the anteroposterior dimension of the nasopharyngeal sphincter following midface distraction, but in practice this is not common. In our series, only two patients reported perceived deterioration of their speech after distraction, while all others felt that there was no difference or even improvement in their intelligibility following distraction. There have been papers which have attempted to predict deterioration in the velopharyngeal function following distraction by measuring the levator activity in the predistraction phase, but the results have been inconsistent.[22]

## CONCLUSION

Distraction osteogenesis is a powerful tool in the armamentarium of the cleft surgeon for the correction of midface deficiencies. However, it needs careful patient selection since growing children may need further orthognathic corrections at the completion of growth. It also needs meticulous planning and follow-up and the potential complications need to be anticipated, identified early, and corrected during the course of treatment. Relapse can be minimized with overdistraction and prolonged use of retention devices.
